# Health professional’s job satisfaction and its determinants in Ethiopia: a systematic review and meta-analysis

**DOI:** 10.1186/s13690-021-00664-7

**Published:** 2021-08-05

**Authors:** Bekahegn Girma, Jemberu Nigussie, Alemayehu Molla, Moges Mareg

**Affiliations:** 1 Department of Nursing, College of Medicine and Health Science, Dilla University, Dilla, Ethiopia; 2 Department of Psychiatry, College of Medicine and Health Science, Dilla University, Dilla, Ethiopia; 3 Department of reproductive health, School of Public Health, College of Medicine and Health Science, Dilla University, Dilla, Ethiopia

**Keywords:** Health professionals, Job satisfaction, Determinants, Ethiopia, Meta-analysis

## Abstract

**Background:**

Health professional’s job satisfaction is directly related to patient satisfaction and quality of care. Without satisfied health professionals the health system is not functional, and the national and global health related plans are not achieved. However, little is known on the level of health professional’s job satisfaction in sub Saharan African countries including Ethiopia. In addition, in Ethiopia there is no summarized evidence helped us an input to design strategies. Therefore, we aimed to assess the pooled prevalence of health professional’s job satisfaction and its determinants in Ethiopia.

**Methods:**

Articles were searched from PubMed, PsycINFO, Hinari, Science Direct, web of science and African journal of online (AJOL) databases, Google and Google scholar. A standardized Microsoft excel spread sheet and STATA software version 16 were used for data extraction and analysis respectively. We followed the Preferred Reporting Items for Systematic reviews and Meta-Analysis to write this report. A random effect meta-analysis model was used to determine the pooled prevalence of job satisfaction. I^2^ was done to check heterogeneity. Egger’s test and funnel plot were conducted to detect publication bias. Subgroup analysis was also conducted. Association was expressed through pooled odd ratio with a 95% CI.

**Result:**

In this review and meta-analysis, a total of 29 studies were included. The pooled prevalence of health professional’s job satisfaction was 46.17% [95% CI (43.08, 49.26)]. The heterogeneity and publication bias test results were I^2^ = 87.3%, *P* <  0.001 and Eggers’, *P* = 0.16. Female sex; OR: 2.20 [95% CI (1.63, 2.97)], working environment; OR: 9.50 [95% CI (6.25, 14.44)], opportunity for professional growth and development; OR: 5.53 [95% CI (1.56, 19.56)], staff relationship; OR: 3.89 [95% CI (1.65, 9.17)] and supportive supervision; OR: 5.32 [95% CI (1.77, 15.92)] were associated with health professional’s job satisfaction.

**Conclusion:**

More than half of professionals were dissatisfied with their jobs. Therefore, the ministry of health and stakeholders better to design strategies to increase the level of satisfaction. Furthermore, it is better to strengthen staff relationship and making the working environment more attractive and equipped.

**Supplementary Information:**

The online version contains supplementary material available at 10.1186/s13690-021-00664-7.

## Background

Job satisfaction is a positive, favourable attitude and feeling what people have about their job [1, 2]. Health professional’s job satisfaction is significantly related with quality of care and patient satisfaction [3]. To achieve the Sustainable Development Goals (SDGs) strategy was planned on human resources by World Health Organization (WHO) [4]. Furthermore, motivation of the health care providers has a pivotal role for achievement of this strategy and it depends on many factors. Job satisfaction is one of the most important factors [5].

A high level of job satisfaction has a positive effect on workers’ health related quality of life [6, 7], job performance [8–10], retention in work [11], quality of healthcare delivery [12, 13] and patient satisfaction [14, 15]. However, low satisfaction may result in staff turnover, tiredness, absenteeism, intention to leave, burnout, undesirable job performance and poor quality of service to clients [16–19].

Shortage of skilled health professionals is a global burden and it is high in most African countries including Ethiopia [20]. According to WHO report by the year 2035 the health workforce shortage will reach up to 12.9 million; 47 and 25% of the shortfall will be in South-East Asia and Africa [21]. To prevent this challenge the health profession’s intention to stay should be increased by increasing their job satisfaction level [22]. Therefore, determining the level of job satisfaction has paramount significance. Studies conducted in Africa on job satisfaction showed a prevalence that ranges from 17.4 [23] to 82.6% [24].

Previous studies identified some determinants for health professional’s job satisfaction such as sex, age, educational status, year of experience, working environment, workload, salary, recognition and opportunity for professionals growth and development [25–32].

In Ethiopia, there is no representative and summarized data on health professional’s job satisfaction. The conducted single studies showed inconsistent prevalence that ranges from 31.7 [33] to 74.4% [34]. Besides, there are some contradicting findings on factors associated with job satisfaction. Therefore, this systematic review and meta-analysis was aimed to estimate the pooled prevalence of health professional’s job satisfaction and its determinants in Ethiopia.

## Methods

### Searching strategy

We followed the Preferred Reporting Items for Systematic reviews and Meta-Analysis (PRISMA) guidelines to write this review and meta-analysis [35]. The presence of similar reviews was checked using this link https://www2.le.ac.uk/library/find/databases/p/Prospero. Primary articles were searched from PubMed, PsycINFO, Hinari, Science direct, web of science and African journal of online (AJOL) databases. Moreover, grey literatures were retrieved from Google and Google scholar. The reference list of published articles was searched to recognize other relevant articles that didn’t showed in databases. Searching was restricted to studies conducted from 2010 until 2020 on humans and full English version articles. Probing of primary articles was started on September 20, 2020 and ended on October 5, 2020. We used “prevalence OR magnitude AND health care professionals OR health care providers OR health workers AND Ethiopia” for objective one and “Determinants OR factors OR predictors AND health care professionals OR health care providers OR health workers AND Ethiopia for the second objective as keywords for searching. Published and unpublished articles were searched and included in this systematic review and meta-analysis. Endnote version X6 was used to manage citations and to check duplication of articles.

### Eligibility criteria

BG and JN independently executed eligibility assessment in an unblinded identical manner based on the stated inclusion and exclusion criteria. We solved disagreements by consensus and invitation of the remaining two authors.

### Inclusion criteria

Observational studies conducted between 2010 and 2020 in Ethiopia among health professionals and published in English language were included. Furthermore, articles reporting the level of health professional’s job satisfaction in proportion were included. Published and unpublished full articles reported the prevalence and/ or associated factor were considered.

### Exclusion criteria

Studies qualitative in design were excluded.

### Outcome measures

In this systematic review and meta-analysis two objectives were assessed. The first was to estimate the pooled prevalence of job satisfaction among health professionals in Ethiopia and it was estimated by dividing the number of satisfied health care professionals to the total number of health care professionals included in this review and meta-analysis, and multiplied by 100. The second objective was to determine the pooled effects of factors on health professional’s job satisfaction in Ethiopia. In this systematic review and meta-analysis, variables identified as a factor for job satisfaction in at least three studies were considered. We used odds ratio (OR) to express the pooled effect.

### Quality assessment and data extraction

Newcastle Ottawa Scale was used to assess the quality of studies [36]. BG and JN have appraised the studies independently using the above tool. The following items were included to evaluate the methodological quality, comparability, and the ascertainment of the outcome. The tool encompassed 10 criteria’s for rating different quality elements and studies scored 6 and above out of 10 were included in to this review and meta-analysis. During the quality assessment any disagreements were solved through discussion and before agreement reached weighted kappa index was done, which was 0.76 [37].

BG and JN extracted all the necessary data independently using a standardized Microsoft Excel. Two data extraction formats, one for each objective were used. The first data extraction format that was prepared for objective one (prevalence of job satisfaction) comprised author name, publication year, region the study conducted, study design, sample size, response rate, profession and prevalence of job satisfaction. To extract data for objective two (factors for job satisfaction) we used two by two tables. Any disagreements during the data extraction time between the two authors (BG and JN) were resolved through discussion, two fold checking the varying data together, and through invitation of either of the remaining two authors.

### Publication bias and heterogeneity

To check publication bias funnel plots [38] and Egger’s statistical test [39] were conducted. We used a *p*-value < 0.05 to declare the statistical significance of publication bias. After a comprehensive examination of the included studies, I^2^ test was conducted to assess the heterogeneity and declared as low, moderate, and high heterogeneity if it is < 50, 50–75%, and > 75% respectively [40].

### Statistical method and analysis

All extracted data from each study using a Microsoft excel spread sheet were exported to STATA version 16 software for analysis. Binomial distribution formula was used to calculate the standard error of prevalence for each original article. Due to high heterogeneity between the included studies we used a random-effect model for analysis [41]. Subgroup analysis based on profession type and region was conducted to check the source of heterogeneity [42–44]. Using separate groups of meta-analysis the effect of the selected associated factors on the outcome variable was examined. We used texts, tables, forest plots, and OR with 95% confidence intervals (CI) to describe the features of the included articles and to display the finding of this review and meta-analysis.

## Results

### Study search and selection

**Fig. 1:**
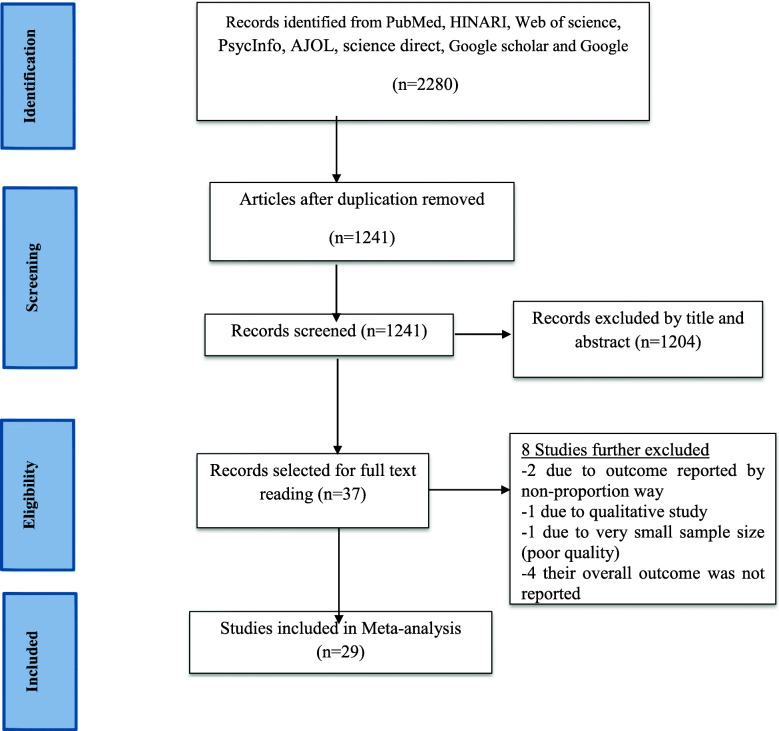
Flow diagram of the studies included in the review of health professionals job satisfaction in Ethiopia, 2020

By fixing the searching scope on full-text articles, human studies, published in English language between 2010 & 2020, we found a total of 2280 primary articles from PubMed HINARI, web of science, PsycInfo, AJOL and science direct databases. Furthermore, we also found from Google scholar and Google. Of which, a total of 1039 and 1204 articles were excluded due to duplication and unrelated to our study that was screened by title and abstract respectively. In this systematic review and meta-analysis, only 37 articles were selected for full reading and 8 were excluded; 4 due to the overall job satisfaction level was not reported [45–48], 1 due to poor quality, since very small sample size used [34], 2 articles due to the outcome variable was not reported with proportion [49, 50] and 1 due to it was qualitative study [51]. Lastly, a total of 29 articles that fulfill all the inclusion criteria’s were selected for meta-analysis [16, 33, 52–78] (Fig. 1).

### Characteristics of the included studies

All of the included articles were cross-sectional studies. The study was conducted among 8368 health care professionals working in Ethiopia. The least sample size was on study done at Mekelle (60) [62] and the highest was in Amhara region (657) [33]. Only three articles were conducted before 2015 [16, 54, 55]. The majority, 9 (30%) of studies were conducted in Oromia region [16, 52, 54, 62, 68, 69, 72, 75, 76] and only one study was conducted in SNNP region [55]. Simple random sampling technique was used by sixteen (53.3%) studies to select study participants [33, 52, 55, 56, 59–61, 65–69, 71–73, 76]. In this review, the response rate of the included articles was ranged from 84.8 [57] to 100% [69]. The highest prevalence of job satisfaction was reported on study done in Mekelle city (67.3%) [62] and the least was in Amhara region (31.7%) [33]. Regarding to profession, 14 (46.7%) were conducted by including all health professionals [16, 33, 63, 65, 66, 68–70, 72, 74–78] and 4 on anaesthesia and midwiferies; 2 for each [61, 64, 68, 71] (Table 1).

**Table 1:** Characteristics of the included articles for health professional’s job satisfaction in Ethiopia; 2020 (*n* = 29)

Author’s	Region	Design	Sample size	Response rate	Prevalence	Profession
Temesgn K et al	Amhara	CS	657	87.5	31.7	Health professionals
Deriba BK et al	All	CS	322	95.56	41.46	Health professionals
Teka AA et al	Oromia	CS	305	100	34.4	Health professionals
Workineh I et al	Oromia	CS	429	98.3	46	Health professionals
Geleto A et al	Harare	CS	420	96.43	44.2	Health professionals
Ayalew F et al	All	CS	500	84.8	60.8	Nurse
Belay YB	Tigray	CS	60	91.6	67.3	Pharmacist
Ayele Y et al	All	CS	232	94.8	32.7	Pharmacist
Belay A et al	Oromia	CS	98	100	48	Nurse
Wondwossen Y et al	AA	CS	314	95.5	37	Health professionals
Kibwana S et al	All	CS	252	100	42.5	Anaesthetics
Asegid A et al	SNNP	CS	278	87	52.5	Nurse
Meselu BT et al	Tigray	CS	140	100	43.57	Midwifery
Salgedo WB et al	Oromia	CS	260	100	49.6	Health professionals
Desalegn N et al	Ethiopia	CS	265	91.3	45.8	Anaesthetics
Kitaba M et al	All	CS	417	94.7	51.8	Nurse
Yami A et al	Oromia	CS	160	90.6	41.4	Health professionals
Mengistu MM et al	Oromia	CS	166	100	34.9	Health professionals
Fentie DY et al	Amhara	CS	104	94.3	46.9	Health professionals
Ahmed SM et al	Oromia	CS	102	95	60.8	Pharmacist
Abadiga M et al	Oromia	CS	266	94.7	51.3	Nurse
Haile D et al	Amhara	CS	181	98.3	54.2	Nurse
Gedif G et al	Amhara	CS	416	92.1	54	Health professionals
Bekru ET et al	AA	CS	234	94.4	52.9	Midwifery
Azagew AW et al	Amhara	CS	416	97.6	49.8	Nurse
Ayalew E et al.	Amhara	CS	226	98.3	43.6	Nurse
Mohammed E et al	Oromia	CS	422	92.2	47.6	Health professionals
Tadesse T et al	AA	CS	304	96.4	43.2	Health professionals
Merga H et al	All	CS	422	98.3	38.5	Health professionals

**Hint:** All: studies conducted county wide; *AA* Addis Ababa; *CS* Cross sectional; Eastern Ethiopia: include Harare and Dire Dawa; *SNNP* South Nation Nationalities and people

### Meta-analysis

**Fig. 2:**
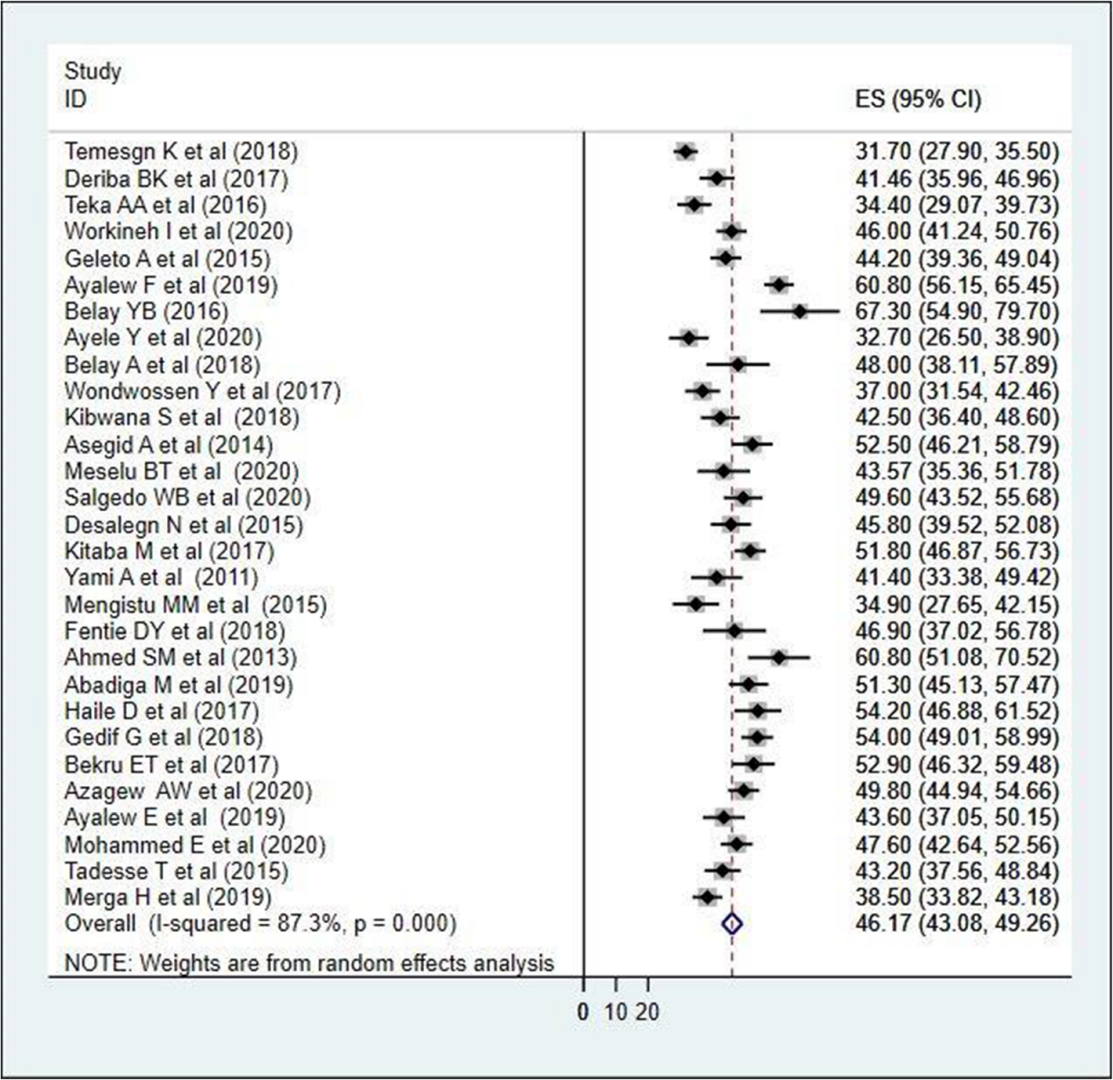
Forest plot of the included studies to determine the pooled prevalence of health professional’s job satisfaction in Ethiopia, 2020 (*n* = 29)

**Fig. 3:**
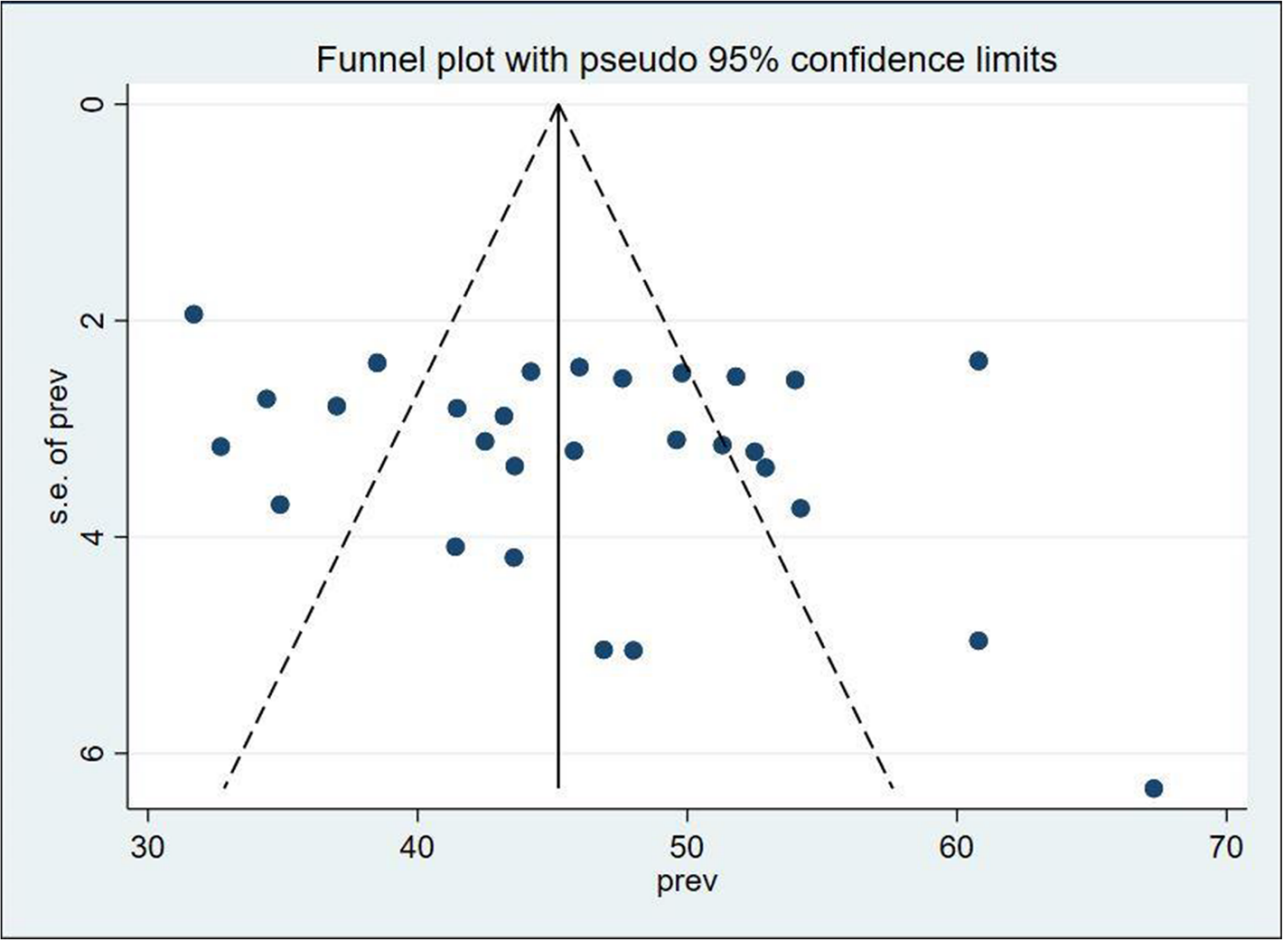
Funnel plot showing distribution of studies on health professional’s job satisfaction in Ethiopia, 2020 (*n* = 29)

As shown on Fig. 2, forest plot was conducted to show the result of the included studies. Twenty nine studies were included in this systematic review and meta-analysis to estimate the pooled prevalence of health care professional’s job satisfaction. The heterogeneity of the included studies was I^2^ = 87.3% with *p* <  0.01. Due to this heterogeneity, we used the random-effect model to estimate the pooled prevalence of health professional’s job satisfaction and it was 46.17%; [95% CI (43.08, 49.26)]. Based on the subjective inspection of funnel plot, more than half of the studies were distributed within the plot (Fig. 3). In addition, Eggers test (*P* = 0.16) was also done, so we have no evidence for publication bias.

To detect the source of heterogeneity subgroup analysis was conducted based on profession and publication year. As shown in Table 2, the highest pooled prevalence of job satisfaction and heterogeneity were shown among pharmacy professionals 53.18 (30.14, 76.22) (I^2^ = 94.7, *P* <  0.01). However, there was no significant pooled prevalence difference among studies conducted before 2017, and 2017 and after; 46.24 (40.55, 51.93) and 46.14 (42.39, 49.90) respectively.

**Table 2:** Sub group pooled prevalence of health professional’s job satisfaction in Ethiopia; 2020 (*n* = 29)

Variables	Characteristics	No. of studies	Prevalence	Heterogeneity	
				I^**2**^ (%)	***P*** value
Publication year	2017 and after	20	46.14 (42.39, 49.9)	88.6	< 0.01
	Before 2017	9	46.24 (40.55, 51.93)	84.4	< 0.01
Profession	Health professionals	14	42.12 (38.45, 45.80)	84.2	< 0.01
	Nurse	8	51.83 (48.07, 55.58)	67.8	< 0.01
	Pharmacist	3	53.18 (30.14, 76.22)	94.7	< 0.01
	Anaesthetics	2	44.10 (39.73, 48.48)	0	0.46
	Midwifery	2	48.57 (39.45, 57.69)	66.9	0.08
Overall pooled prevalence		29	46.17 (43.08, 49.26)	87.3	< 0.01

### Factors associated with health professional’s job satisfaction

In the present review and meta-analysis, factors identified as significant for health professional’s job satisfaction in at least three primary articles were included. As a result, salary [53, 60, 65, 75], working experience [33, 55, 67–69, 76] and profession type [33, 66, 69] were identified as factors for job satisfaction in three and above studies, due to they were reported through various category methods that create a difficulty to extract data we exclude them from analysis after discussion and agreement by authors. Support from supervisor [65, 73, 76], working environment [56, 60, 66, 72, 73, 76], opportunity for professional growth [60, 74, 76], sex [61, 69, 73], marital status [58, 61, 65], educational status [33, 61, 69, 76], staff relationship [72, 74, 76] and age [59, 69, 76] were factors that fulfill the criteria and selected for meta-analysis. However, support from supervisor, working environment, staff relationship, sex and opportunity for professional growth were identified as significant determinants for health professional’s job satisfaction.

In the current review and meta-analysis, health professionals who had good support from their supervisors were 5.3 times more likely to satisfy as compared to their contrary health care professionals (OR: 5.32 [95% CI (1.77, 15.92)]). Health professionals who were worked in an attractive or good environment had 9.5 times higher opportunity to satisfy in contrast to their counterparts (OR: 9.50 [95% CI (6.25, 14.44)]). Moreover, health professionals who were worked in an organization that provide opportunity for professional growth and development like training and education access had 5.5 times higher satisfaction level than those working in an organization that doesn’t provide any opportunity for professional growth and development (OR: 5.53 [95% CI (1.56, 19.56)]) (Table 3).

**Table 3:** Meta-analysis of factors associated with health professional’s job satisfaction in Ethiopia, 2020

Factors	Number of studies	OR (95% CI)	Heterogeneity	
			I^**2**^ (%)	***P*** value
Support from supervisor	3	5.32 (1.77, 15.92)^**a**^	94.3	< 0.01
Condition of working environment	6	9.50 (6.25, 14.44)^**a**^	76.0	< 0.01
Opportunity for professional growth	3	5.53 (1.56, 19.56^**a**^	95.2	< 0.01
Sex	3	2.20 (1.63, 2.97)^**a**^	0	0.42
Marital status	3	1.07 (0.66, 1.75)	68.5	0.04
Educational status	4	1.70 (0.58, 5.00)	93.7	< 0.01
Staff relationship	3	3.89 (1.65, 9.17)^**a**^	88.1	< 0.01
Age	3	0.73 (0.37, 1.44)	67.4	0.05

^**a**^significantly associated factors

As shown below in Table 3, staff relationship and sex were another factor identified for health professional’s job satisfaction. Health professionals who were worked in an organization that had good relationship between staffs and female in sex had 3.8 (OR: 3.89;[95% CI (1.65, 9.17)]) and 2.2 times (OR: 2.20 [95% CI (1.63, 2.97)]) more chance to satisfy as compared to their opposite groups respectively.

## Discussion

Job dissatisfaction worsen patient-health professionals ratio and staff turnover [22]. Job satisfaction is strongly associated with workers depression and anxiety [6]. The aim of this review and meta-analysis was to assess the pooled prevalence of health professional’s job satisfaction and its determinants in Ethiopia. The pooled prevalence of health professional’s job satisfaction was 46.17% [95% CI (43.08, 49.26)]. We used random effect model for pooled analysis of included studies, since heterogeneity was 87.3% and the Egger test value was 0.06 before excluding one single study done in Harare region with very small sample size (43) [34] and after excluding this single study the value of Egger test was 0.16. While inspecting funnel plot more than half of the articles were within the plot.

Support from supervisor, working environment, staff relationship, sex and opportunity for professional growth were identified as significant determinants for health professional’s job satisfaction.

In this review and meta-analysis, the overall pooled prevalence of job satisfaction was lower as compared to studies conducted in South Africa (52.1%), Malawi (71%), Tanzania (82.6%) [24], Canada 56% [79], Nepal (76%) [26], Spain (77.2%) [80], India (59.6%) [81], Egypt (61.24%) [82], China (74.6%) [3] and the global population job satisfaction level (88%) [83]. This might be due to variation in socio-economic, health facility quality and payment for health professionals. However, it was high as compared to studies done in Pakistan (41%) [84], Nigeria (32.9%) [85] and Uganda (17.4%) [23]. The discrepancy might be because of study population variation that the last two were conducted on only nurses and the second reason might be due to small sample size in the above studies.

Health professionals who had good support from their supervisors were more likely satisfied. This finding was supported by study done in Pakistan [84] and studies conducted in three African countries [86, 87]. This might be due to the fact that good support from supervisors increases professional’s motivation that leads to satisfaction. In addition it might be due to the fact that supportive supervision increase staff job satisfaction level [88].

Working environment of the employees was significantly associated with job satisfaction. Health professionals who were working in an attractive and good area had 9.5 times more likely satisfied as compared to their counterparts. This finding was supported by qualitative study conducted in Pakistan [89]. This might be the fact that good and attractive working environment have positive psychological and physical influence on employees and motivate them.

In the current review and meta-analysis, health professionals who were working in an organization that had good staff relationship were more satisfied. This finding was supported by studies done in India [90] and Bangladesh [91]. This similarity might be due to good staff relationship create good communication between staffs that leads to good working condition and satisfaction.

Employees who were working in an organization which provide an opportunity for professional growth and development had 5.5 times more odds of satisfaction. This finding was supported by studies conducted in South Africa [92] and Nepal [26]. This might be due to the fact that such kind of opportunity increase professional’s income, level of knowledge and motivation that lead to satisfaction with their job. Moreover, it might be due to the fact that professional development have a direct positive effect on job satisfaction [93].

The last determinant identified in this review and meta-analysis was sex. Female health professionals had more satisfied as compared to their counterparts. This finding was supported by studies done in China [3] and USA [94]. This might be because of the fact that female doesn’t express their dissatisfaction openly rather internalizing the feeling of misery [95].

The first limitation of this study might be difficulty of establishing temporal relationship between the outcome and independent variables since all the included articles were cross sectional studies. Second, the presence of heterogeneity might be the indicative for the existence of underling effect among primary studies. Third, searching is limited to Ethiopia as result the evidence might not be generalized to other countries in the world.

## Conclusion

In this review and meta-analysis, more than half of health professionals were dissatisfied with their job. Good support from supervisor, working environment, staff relationship, female sex and availability of opportunities for professional growth and development were associated with health professional’s job satisfaction. In order to increase the level of job satisfaction the federal ministry of health and stakeholders better to develop a strategy and strength staff relationship, support from supervisors, professional growth opportunities and make the working environment more attractive.

## Supplementary Information

**Additional file 1.**

## Data Availability

The data included in this study is available and can be accessed by contacting the corresponding author through this email address; bekahegngi@gmail.com or Bekahegng@du.edu.et.

## Abbreviations

CI: Confidence interval
OR: Odd ratio
SNNP: South nations and nationalities people
SDG: Sustainable development goals
USA: United States of America
WHO: World health organization
