# Supplementary material for: Health professional’s job satisfaction and its determinants in Ethiopia: a systematic review and meta-analysis
Source: Arch Public Health. 2021 Aug 5;79:141. doi: 10.1186/s13690-021-00664-7 (PMC8340440; doi:10.1186/s13690-021-00664-7)
Supplement: Supplementary file 1 — Additional file 1. [file 13690_2021_664_MOESM1_ESM.docx]

**Supplementary information**

It contains the results of subgroup analysis

1. Sub group analysis by region

1. Subgroup by profession

1. Subgroup analysis by publication year
